# Norms of assertion in the United States, Germany, and Japan

**DOI:** 10.1073/pnas.2105365118

**Published:** 2021-09-10

**Authors:** Markus Kneer

**Affiliations:** ^a^Centre for Ethics, Department of Philosophy, University of Zurich, 8008 Zurich, Switzerland

**Keywords:** communication, assertion, norms, misinformation

## Abstract

The recent controversy about misinformation has moved a question into the focus of the public eye that has occupied philosophers for decades: Under what conditions is it appropriate to assert a certain claim? When asserting a claim that *x*, must one know that *x*? Must *x* be true? Might it be normatively acceptable to assert whatever one believes? In the largest cross-cultural study to date (total *n* = 1,091) on the topic, findings from the United States, Germany, and Japan suggest that, in order to claim that *x*, *x* need not be known, and it can be false. However, the data show, we do expect considerable epistemic responsibility on the speaker’s behalf: In order to appropriately assert a claim, the speaker must have good reasons to believe it.

Human action and interaction are heavily governed by conventions, norms, and laws. Over the last few decades, philosophers have explored whether assertion—the backbone of linguistic communication, and thus all language-dependent human practices—is regulated by norms. The topic could not be more pertinent to the current misinformation controversy that shapes public discourse in the United States and many other countries (1), which has intensified since the COVID-19 pandemic (2).

Assertions are speech acts by aid of which we share information (3). What makes it appropriate or inappropriate for a speaker to assert a claim with the content or proposition *x*? The field is roughly divided into two main camps (4). “Factivists” argue that one should make an assertion only if its content is at least true. Some limit themselves to truth [the “truth account” (5)]. Others insist that, in order to assert that *x,* one must know that *x*, that is, (roughly) have a true, justified belief that *x* [the “knowledge account” (6–8)]. Naturally, the more demanding the epistemic requirements on the part of the agent, the smaller the number of warranted assertions. This is what inspires “nonfactivists” to hypothesize that it is acceptable to assert a proposition for which one has good evidence [the “justified belief account” (9, 10)], although it might turn out to be false. More lenient nonfactivists, of which there are few, would predict that it is fine for a speaker to assert whatever she in fact believes, regardless of whether the belief is well justified [the “belief account” (11, 12)]. According to this view, the norm of assertion is simply a matter of refraining from lying.

Philosophy might be suited to elucidate the nature, function, or aim of assertion (13). Which norm, if any, in fact governs the practice of assertion, however, is an empirical question (3, 9). Some empirical findings are consistent with a factive norm of assertion (see ref. 8 for an overview); others are more in tune with the nonfactive justified belief account (14–16). Nearly all work to date has focused on American native English speakers, although there are some data for Korean speakers (17). The studies presented here explore the norm of assertion among native speakers of German and Japanese (in their languages), so as to compare the findings with those for American English speakers.

The paper pursues a two-step procedure (Fig. 1): Study 1 explores whether the norm of assertion in the target countries is factive. If it were, a second study would have to investigate whether true belief, by itself, suffices, or whether assertibility requires—as many philosophers believe—knowledge. The other possibility is that the results of study 1 show that truth is not required for a claim to be assertible. This is indeed what we find. Consequently, study 2 has to adjudicate between the two nonfactive accounts of the norm of assertion: justified belief vs. mere belief. In all three countries, it turns out, the norm of assertion is justified belief.

**Fig. 1.**
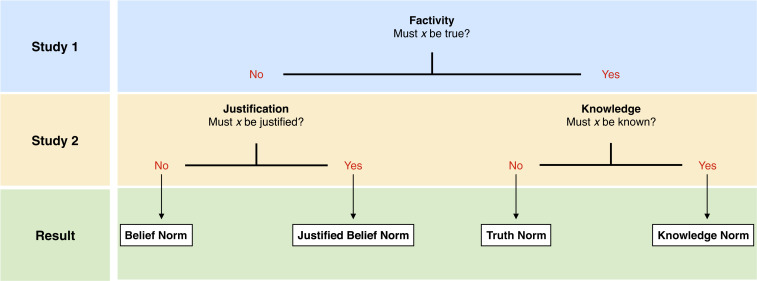
Procedure for the empirical exploration of norms of assertion.

## Results

Study 1 (*n* = 495) used a classic vignette from epistemology in which Bob has good reason to believe that his colleague Jill drives an American car. In one condition, Jill still drives an American car (true justified belief); in the other, she has changed to a German car (false justified belief). Participants were randomly assigned to one of the two conditions. They were asked whether Bob, when prompted, 1) should say that Jill drives an American car (assertibility) and, as a manipulation check, 2) whether it is true that Jill drives an American car (truth). The questions used a forced-choice Yes/No response mechanism.

**Fig. 2.**
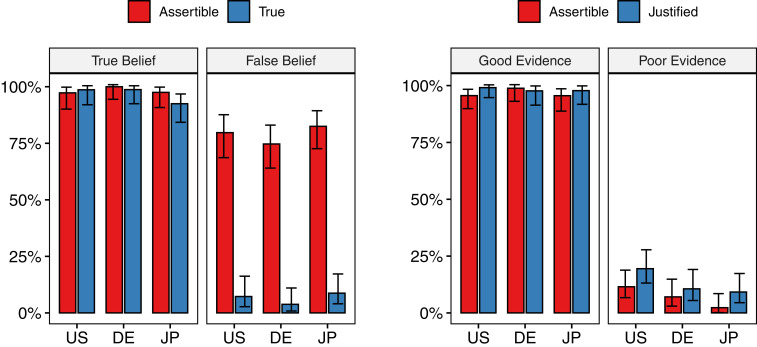
(*Left*) Study 1: Proportions of participants who judged *x* assertible and the proposition *x* as true across conditions (true vs. false belief) and countries (United States vs. Germany vs. Japan). (*Right*) Study 2: Proportions of participants who judged *x* assertible and the belief of *x* as justified across conditions (good evidence vs. poor evidence) and countries.

Advocates of a factive norm of assertion expect low rates of assertibility in the false belief condition. Advocates of a nonfactive norm predict high rates of assertibility in the false belief condition, since Bob’s belief is nonetheless justified. The latter prediction turned out to be correct (Fig. 2, *Left*): Although truth did have some impact on assertibility (χ^2^(1, *n* = 461) = 43.05, *P* < 0.001, ϕ = 0.31), at least three out of four participants considered it appropriate to assert a false justified belief (United States: 80%; Germany: 75%; Japan: 83%; all significantly above chance, binomial tests, *P* < 0.001, all two-tailed). Country was nonsignificant (χ^2^(2, *n* = 461) = 0.56, *P* = 0.755, ϕ = 0.035). In short, the question as to whether the assertibility of a claim requires truth was answered with a resounding “no” across all three cultures and languages tested. Since knowledge entails truth, there is no need to investigate the factive accounts further. Study 2 thus explored the nonfactive accounts (Fig. 1, left), testing whether assertibility requires justified belief, or whether it is acceptable to assert whatever one believes.

Study 2 (*n* = 596) employed the “airport scenario” from ref. 14, in which a lady at the airport asks Carlos which gate the flight to Paris departs from. In one condition, Carlos finds the flight in the departure list (good evidence). In the other condition, he cannot find the flight in the list, but has a hunch that it will depart from gate 24 (bad evidence). In either case, Carlos says that the flight leaves from gate 24. Participants were randomly assigned to one of the two conditions and asked whether Carlos’ claim was assertible and, as a manipulation check, whether his belief was justified (forced-choice Yes/No for both questions).

Assertibility was high when the speaker had good reasons for the asserted claim (United States: 96%; Germany: 99%; Japan, 96%; all significantly above chance, binomial tests, all *P* < 0.001); see Fig. 2, *Right*. Assertibility was low when the speaker did not have good reasons (United States: 12%; Germany: 7%; Japan: 2%; all significantly below chance, binomial tests, all *P* < 0.001). A regression analysis (χ^2^(5, *n* = 575) = 568.09, *P* < 0.001, Nagelkerke *R*^2^ = 0.84) revealed justification to be a significant and powerful predictor of assertibility (*P* < 0.001; odds ratio: 913.75). Country was nonsignificant (*P* = 0.437), and the same held for the interactions (all *P* > 0.100). Inconsistent with some previous findings for the United States (8), although consistent with others (14–16), the norm of assertion seems to be justified belief—and the same holds for Germany and Japan.

## Discussion

Under what conditions is it appropriate for a speaker to assert a certain belief? According to the dominant philosophical view, one should only say that *x* if one knows that *x* (refs. 6–8; see also refs. 3 and 4). In an experiment run in three countries and languages, we found that this account is incorrect: When held for good reasons, false beliefs are deemed nearly as assertible as true beliefs. Consistent with some previous research (14–16), this suggests that neither truth nor knowledge constitute the norm of assertion. Do the findings suggest that one can thus simply assert what one believes? Study 2 demonstrates that this is not the case, and the results are once again near-identical across all three cultures: In order to be in a position to assert a proposition *x*, one must have good reasons for believing *x*.

Despite the decisive nature of the results and their similarity across cultures, it is, of course, too early to postulate a universal norm of assertion. More research is needed, *inter alia*, in small-scale societies, whose general normative fabric can differ considerably from Western, educated, industrialized, rich, and democratic countries (18). At least for the countries tested, however, the findings provide some important insights concerning the misinformation controversy (1, 2): What people object to is not, strictly speaking, the dispersion of false claims but, rather, unjustified claims through which the speaker manifests a certain disregard for truth.

## Extended Methods

The studies were conducted with approval from the Ethics Commission of the University of Zurich. All participants provided informed consent to take part in the online survey.

### Study 1.

#### Participants.

For study 1, 495 participants were recruited in the United States, Germany, and Japan via crowdworking platforms (details in *SI Appendix*). As preregistered, inattentive subjects, those spending less than 10 s on the main task (reading the short scenario and answering the assertibility question), and nonnative speakers of English, German, or Japanese, respectively, were excluded. The final datasets comprised 143 subjects for the United States (73 female, age M = 38 y, SD = 11 y), 158 subjects for Germany (96 female, age M = 40 y, SD = 12 y), and 160 subjects for Japan (83 female, age M = 41 y, SD = 10 y).

#### Results.

As a further manipulation check, people were asked whether the protagonist’s belief was justified. As intended, the vast majority of participants attributed justification in both conditions in all three countries (significantly above chance, binomial tests, all *P* < 0.001). As concerns the main analysis, due to a cell count of zero for “unassertible” in the German true belief condition (which is consistent with the hypotheses of all accounts), a logistic regression analysis could not be performed. However, a logistic regression with the full sample (no exclusions, which did not change the results) was possible and is reported in *SI Appendix*, Table S3. Consistent with the Pearson’s Chi Square results, country had no impact on assertibility (*P* = 0.423). There was some impact of truth value (*P* = 0.007, odds ratio = 0.123), although the model explained only about 20% of the variance (Nagelkerke *R*^2^ = 0.196). All interactions were nonsignificant (all *P* > 0.227).

### Study 2.

#### Participants.

For study 2, 596 participants were recruited in the United States, Germany, and Japan via crowdworking platforms. As preregistered, participants failing an attention check or a comprehension check and nonnative speakers of the three languages were excluded. There were 227 participants from the United States (111 female, age M = 44 y, SD = 12 y), 171 from Germany (108 female, age M = 38 y, SD = 12 y), and 177 from Japan (91 female, age M = 40 y, SD = 10 y).

#### Results.

To increase external validity, the formulation of the assertibility question was also manipulated: It either asked whether Carlos “should have said” or whether it “was appropriate for Carlos to say” that the flight leaves from gate 24. Due to a cell count of zero in the justified belief condition in certain countries, formulation could not be entered as a predictor into the regression analysis, but a Pearson Chi Square test revealed formulation to be nonsignificant (χ^2^(1, *n* = 575) = 0.52, *P* = 0.469, ϕ = 0.030). Besides the manipulation check on justification, a second check was run to ensure that participants understood that the protagonist did indeed believe the proposition at issue and thus was not interpreted as lying. As intended, at least about four in five participants ascribed belief in all conditions in all three countries (significantly above chance, binomial tests, all *P <* 0.001).

### Materials.

For both studies, the materials (in English, German, and Japanese) and detailed analyses are reported in *SI Appendix*. *SI Appendix*, the preregistrations, the Qualtrics files, and the data are deposited on the Open Science Framework (OSF) page: https://doi.org/10.17605/OSF.IO/H6M49.

## Supplementary Material

Supplementary File

## Data Availability

Anonymized, complete datasets have been deposited on OSF (https://doi.org/10.17605/OSF.IO/H6M49) (19).
