# Supplementary material for: Norms of assertion in the United States, Germany, and Japan
Source: Proc Natl Acad Sci U S A. 2021 Sep 10;118(37):e2105365118. doi: 10.1073/pnas.2105365118 (PMC8449385; doi:10.1073/pnas.2105365118)
Supplement: Supplementary File [file pnas.2105365118.sapp.pdf]

Norms of assertion in the US, Germany and Japan
A cross-cultural exploration of what we expect from each other in linguistic communication

Markus Kneer
University of Zurich
markus.kneer@uzh.ch

Appendix

1. Study 1

1.1 Participants
A total of 495 participants were recruited on online platforms in the US (Amazon Mechanical Turk), Germany (Clickworker) and Japan (Crowdworks). As preregistered,[1] participants whose native language was not English, German or Japanese respectively, and those who failed an attention or comprehension check were excluded, leaving 461 participants (252 female, age M=40 years, SD=11 years). The subsamples for each country were similar in terms of gender distribution and average age.[2]

1.2 Materials
1.2.1 Materials in English
The vignette, originally from Nichols, Stich & Weinberg (2003) and previously used in experiments regarding the norm of assertion by Kneer (2018) and Turri (2018), had two conditions: One in which the agent had good evidence for a belief which is true, and one in which the agent's belief, although well-justified, is false (variations in square brackets):

> Bob has a friend, Jill, who has driven a Buick for many years. A Buick is an American car. Bob therefore thinks that Jill drives an American car. [He is not aware, however, that Jill regularly cleans its chrome rims with a non-abrasive cloth. / He is not aware, however, that her Buick has recently been stolen, and he is also not aware that Jill replaced it with a Mercedes, which is a German car.]
>
> One day Bob's wife asks him: 'Does your friend Jill drive an American car?'
> Q1: Should Bob say 'Jill drives an American car.'? (Yes/No)
> Q2: Is it true that Jill drives an American car? (Yes/No)
> Q3: Is Bob justified in believing that Jill drives an American car? (Yes/No)

Participants were randomly assigned to one of the two conditions.

[1] https://aspredicted.org/blind.php?x=rv7km4
[2] US: Total 143 (170 pre-exclusions), 73 female, age M=38 years, SD=11 years. Germany: Total 158 (164), 96 female, age M=40 years, SD=12 years. Japan: Total 160 (161), 83 female, age M=41 years, SD=10 years.

1.2.2 German Translation

Mark hat eine Freundin, Kathrin, die seit vielen Jahren einen Audi fährt. Ein Audi ist ein deutsches Auto. Mark denkt daher, dass Kathrin ein deutsches Auto fährt. [Er weiß allerdings nicht, dass Kathrin die Chromfelgen regelmäßig mit einem nicht scheuernden Tuch reinigt. / Er weiß allerdings nicht, dass ihr Audi vor kurzem gestohlen wurde, und er weiß auch nicht, dass Kathrin ihn durch einen Chevrolet ersetzt hat, bei dem es sich um ein amerikanisches Auto handelt.]

Eines Tages fragt Marks Frau ihn: "Fährt deine Freundin Kathrin ein deutsches Auto?"
F.1: Sollte Mark sagen “Kathrin fährt ein deutsches Auto.”? (Ja/Nein)
F.2: Stimmt es, dass Kathrin ein deutsches Auto fährt? (Ja/Nein)
F.3: Ist Marks Überzeugung, dass Kathrin ein deutsches Auto fährt, gerechtfertigt? (Ja/Nein)

1.2.3 Japanese Translation

ボブには、ジルという友人がいます。ジルは長年、アメリカ車である「ビュイック」に乗っています。そのため、ボブはジルがアメリカ車に乗っていると思っています。[ところで、ジルは車のタイヤホイールを定期的に柔らかい布できれいにしているのですが、ボブはこのことに気づいていません。/ところが、ジルのビュイックは最近、何者かに盗まれてしまい、ジルは「メルセデス」というドイツ車を購入しました。ボブは、ジルのビュイックが盗まれてしまったことにも、ジルがメルセデスを購入したことにも気づいていません。]

ある日、ボブの妻が彼に次のように尋ねてきました。「あなたの友人のジルは、アメリカ車に乗っているの？」
問１：ボブは、妻に対して「ジルはアメリカ車に乗っている」と言うべきですか？（はい／いいえ）
問２：ボブの友人のジルがアメリカ車に乗っていることは、真実ですか？（はい／いいえ）
問３：ボブは、友人のジルがアメリカ車に乗っていると信じるに足る理由をもっていますか？（はい／いいえ）

1.3 Results

Factive accounts predict assertability to be low in the false belief condition – at the very least significantly below chance. However, in line with predictions of nonfactivist accounts of assertion, at least 3 out of 4 participants considered it perfectly acceptable to assert a false claim in all three countries (significantly above chance, binomial tests, all *p*s<.001, two-tailed), see Table 1. The results demonstrate quite clearly that the norm of assertion is not factive. Expectedly, in the true belief condition, assertability exceeded 97% in all three countries. Both manipulation checks suggest that the vignettes worked well: In the true belief condition, nearly everyone considered the proposition at stake true (at least 93%, significantly above chance, binomial tests, all *p*s<.001), and hardly anybody considered it true in the false belief condition (at

most 9%, significantly below chance, binomial tests, all *p*s<.001). In both conditions justification was perceived to be high (at least 68%, significantly above chance, binomial tests, all *p*s<.003), although – in both conditions – it was a little lower for Japanese subjects in comparison to US Americans and Germans.

Due to a cell count of zero for "unassertable" in the German true belief condition (which is consistent with the hypotheses of all accounts), a binomial logistic regression analysis could not be performed. Aggregating across conditions, a Pearson Chi Square test revealed no significant difference in assertability judgments across countries (χ^2(2,n=461)=.56, *p*=.755, ϕ=.035). Aggregating across countries, truth did have a significant, though not particularly large, impact on assertability (χ^2(1,n=461)=43.05, *p*<.001, ϕ=.31).

	Assertability			Truth			Justification		
	US	D	JP	US	D	JP	US	D	JP
True Belief	97%	100%	98%	99%	99%	93%	91%	97%	84%
False Belief	80%	75%	83%	7%	4%	9%	91%	85%	68%

Table 1: Proportions of participants who consider *p* assertable, true and believed with justification across conditions (true belief v. false belief) and countries (US v. Germany v. Japan).

Including the excluded participants (less than 10%) in the sample left the results by and large the same (see Table 2). It did, however, make it possible to conduct a regression analysis (we no longer have a cell count of zero for "unassertable" in Germany), see Table 3. Truth had a significant impact on assertability (*p*=.007, Odds Ratio=.123), Country and the interactions were nonsignificant (all *p*s>.227). Although truth was significant, note that the model explained less than 20% of the variance (Nagelkerke R^2=.196).

	Assertability			Truth			Justification		
	US	D	JP	US	D	JP	US	D	JP
True Belief	97%	99%	98%	97%	99%	93%	88%	98%	84%
False Belief	75%	76%	83%	13%	7%	10%	89%	84%	67%

Table 2: Proportions of participants who consider *p* assertable, true and believed with justification across conditions (true belief v. false belief) and countries (US v. Germany v. Japan); full sample (n=495) without exclusions.

	B	*SE*	*Wald*	*df*	*p*	*Odds Ratio*	*LLCI*	*ULCI*
Truth	-2.098	0.774	7.346	1	0.007	0.123	0.027	0.559
Country			1.721	2	0.423			
Country (1)	0.467	0.387	1.456	1	0.228	1.595	0.747	3.407
Country (2)	0.434	0.39	1.237	1	0.266	1.544	0.718	3.319
Truth * Country			0.917	2	0.632			
Truth * Country(1)	-0.124	1.004	0.015	1	0.902	0.884	0.123	6.323
Truth * Country (2)	-1.165	1.295	0.809	1	0.368	0.312	0.025	3.949
Intercept	-1.566	0.294	28.386	1	<.001	0.209		

Table 3: Regression analysis with Truth Value and Country as predictors of assertability. $\chi^2(5, n=495)=53.89$, $p<.001$, Cox & Snell $R^2=.103$, Nagelkerke $R^2=.196$. Reference classes: Truth value = false, Country = Japan. Coding Country (1): US=1, D=0, JP=0; Country (2), US=0, D=1, JP=0. 87.7% of cases were correctly classified.

Aggregating across country (since nonsignificant), a final regression analysis with the full sample used perceived truth and justification as predictors (i.e. people's responses as to whether they considered the proposition true and justified) of assertability. The results (Table 4) show that justification – although *not* manipulated – had a *larger* impact than truth, which was manipulated.

	B	*SE*	*Wald*	*df*	*p*	*Odds Ratio*	*LLCI*	*ULCI*
Truth	-1.509	0.576	6.859	1	0.009	0.221	0.071	0.684
Justification	-1.568	0.366	18.322	1	<.001	0.208	0.102	0.427
Truth*Justification	-0.253	0.721	0.124	1	0.725	0.776	0.189	3.19
Intercept	-0.14	0.306	0.209	1	0.648	0.87		

Table 4: Regression analysis with perceived truth value and perceived as predictors of assertability. $\chi^2(3, n=495)=5.97$, $p<.001$, Cox & Snell $R^2=.111$, Nagelkerke $R^2=.210$. Reference classes: Truth value = false, Justification = unjustified, 87.7% of cases correctly classified.

2. Study 2

2.1 Participants

A total of 596 participants were recruited on online platforms in the US (Amazon Mechanical Turk), Germany (Clickworker) and Japan (Crowdworks). As preregistered,[3] participants whose native language was not English, German or Japanese respectively, and those who failed an attention or comprehension check were excluded, leaving 575 participants (310 female, age M=41 years, SD=12 years). The subsamples for each country were similar in terms of gender distribution and average age.[4]

[3] https://aspredicted.org/blind.php?x=zj4f2w

[4] US: Total 227 (239 pre-exclusions), 111 female, age M=44 years, SD=12 years. Germany: Total 171 (177), 108 female, age M=38 years, SD=12 years. Japan: Total 177 (180), 91 female, age M=40 years, SD=10 years.

2.2 Materials
2.2.1 Materials in English

The experiment, based on Kneer (2018), took a 2 justification (good v. bad evidence) × 2 question formulation ("should have said" v. "appropriate to say") between-subjects design. Participants were randomly assigned to one of the four conditions. In the scenario, justification was manipulated, varying between good evidence and bad evidence (in square brackets):

> Carlos is at the JFK airport in New York, waiting for his flight to Amsterdam. An elderly woman asks him whether he could tell her at which gate the flight to Paris departs. Carlos has a look at the monitor listing the departure gates. [The departure list states that the only flight to Paris leaves at gate 24. / He cannot find the flight to Paris in the list. But Carlos has a hunch that it might depart from gate 24, and thus comes to believe that it will.] Carlos says to the elderly woman: "The flight to Paris leaves at gate 24."

The assertability question came in two variations ("should have said" v. "appropriate to say"). Each participant only saw one of the two assertability questions (i.e. either Q1a or Q1b):

> Q1a: Do you think Carlos should have said that the flight to Paris leaves at gate 24? (Yes/No)
> Q1b: Do you think it was appropriate for Carlos to say that the flight to Paris leaves at gate 24? (Yes/No)
> Q2: Do you think that Carlos's belief that the flight to Paris leaves at gate 24 was justified? (Yes/No)
> Q3: Do you think Carlos believed that the flight to Paris leaves at gate 24? (Yes/No)

2.2.2 German Translation

> Daniel befindet sich am Flughafen in Düsseldorf und wartet auf seinen Flug nach Amsterdam. Eine ältere Frau fragt ihn, ob er ihr sagen kann, an welchem Flugsteig der Flug nach Paris abfliegt. [Daniel wirft einen Blick auf den Monitor, auf dem die Abflugsteige aufgelistet sind. In der Abflugliste steht, dass der einzige Flug nach Paris von Flugsteig 24 abfliegt. / Daniel wirft einen Blick auf den Monitor, auf dem die Abflugsteige aufgelistet sind. Er kann den Flug nach Paris nicht in der Liste finden. Aber Daniel hat ein Gespür, dass er von Flugsteig 24 abfliegen könnte, und glaubt daher, dass dies auch der Fall sein wird.] Daniel sagt der älteren Frau: "Der Flug nach Paris geht von Flugsteig 24."

Q1a: Hätte Daniel Ihrer Meinung nach sagen sollen, dass der Flug nach Paris von Flugsteig 24 abfliegt? (Ja/Nein)
Q1b: War es Ihrer Meinung nach angemessen für Daniel zu sagen, dass der Flug nach Paris von Flugsteig 24 abfliegt? (Ja/Nein)
Q2: War Ihrer Meinung nach der Glaube von Daniel, dass der Flug nach Paris von Flugsteig 24 abfliegt, gerechtfertigt? (Ja/Nein)
Q3: Hat Daniel Ihrer Meinung nach geglaubt, dass der Flug nach Paris von Flugsteig 24 abfliegt? (Ja/Nein)

2.2.3 Japanese Translation

カルロスは、アメリカのニューヨークにあるジョン・F・ケネディ国際空港で、オランダのアムステルダム行きの飛行機を待っていました。そこへ、年配の女性が、フランスのパリ行きの飛行機の出発ゲートを教えてくれないかとカルロスに尋ねてきました。カルロスは、出発ゲートが表示されているモニターを見ました。［モニターには、パリ行きの飛行機は 24 番ゲートからしか出ていないと書かれていました。 / その中には、パリ行きの飛行機は見当たりませんでした。しかし、カルロスはパリ行きの飛行機は 24 番ゲートから出発するのではないかと直感し、パリ行きの飛行機の出発ゲートは 24 番であると考えるようになりました。］カルロスは、女性に次のように言いました。「パリ行きの飛行機は、24 番ゲートから出発しますよ。」

問 1a：カルロスは、年配の女性に対して、パリ行きの飛行機が 24 番ゲートから出発すると言うべきでしたか？（はい / いいえ）
問 1 b：カルロスが年配の女性に対して「パリ行きの飛行機は 24 番ゲートから出発する」と言ったのは、適切だったと思いますか？（はい / いいえ）
問 2：カルロスは、パリ行きの飛行機が 24 番ゲートから出発すると信じるに足る理由をもっていたと思いますか？（はい / いいえ）
問 3：カルロスは、パリ行きの飛行機が 24 番ゲートから出発すると信じていたと思いますか？（はい / いいえ）

2.3 Results

Due to a cell count of zero in several conditions (see Table 5, all consistent with predictions), it was impossible to run a binomial regression analysis with all three factors, i.e. formulation, justification and country. I thus conducted Pearson's Chi Square test, which revealed no significant difference in assertability judgments across formulations (χ^2(1,n=575)=.52, p=.469, ϕ=.030). Averaging across formulations makes it possible to conduct a regression analysis with the two key predictors: justification and country. The analysis revealed a significant and powerful effect for justification (p<.001, Odds Ratio=913.75), see Table 6. Unsurprisingly, the proportions of participants who considered the proposition at stake assertable yet judged it unjustified was significantly below chance in any condition (at most 11%, binomial tests, test

proportion =.25,[5] all *p*s<.001, one-tailed). Overall country, as well as all interactions, proved nonsignificant (all *p*s>.100). The model explained more than 80% of the variance (Nagelkerke R^2=.838). As a manipulation check, participants were consulted as to whether they held that the agent actually believed that *p* in the first place. In any condition in any country at least about four in five participants responded with yes (significantly above chance, binomial tests, all *p*s<.001).

		Assertability			Justification			Belief		
		US	D	JP	US	D	JP	US	D	JP
"Should"	Poor Evidence	7%	9%	0%	20%	9%	11%	86%	93%	84%
	Good Evidence	95%	98%	100%	100%	98%	98%	100%	89%	100%
"Appropriate"	Poor Evidence	17%	5%	5%	19%	12%	7%	89%	100%	79%
	Good Evidence	97%	100%	91%	98%	98%	98%	98%	98%	100%

Table 5: Proportions of participants who consider *p* assertable, justified and believed across formulations ("should" v. "appropriate"), evidential conditions (poor v. good) and countries (US v. Germany v. Japan).

	B	*SE*	*Wald*	*df*	*p*	*Odds Ratio*	*LLCI*	*ULCI*
Justification	6.818	0.879	60.098	1	<.001	913.75	163.026	5121.502
Country			1.657	2	0.437			
Country (1)	-0.014	0.686	0	1	0.984	0.986	0.257	3.785
Country (2)	-1.375	1.128	1.484	1	0.223	0.253	0.028	2.31
Justification * Country			4.141	2	0.126			
Justification * Country (1)	-1.695	1.034	2.688	1	0.101	0.184	0.024	1.393
Justification * Country (2)	0.203	1.402	0.021	1	0.885	1.225	0.079	19.103
Intercept	-3.068	0.511	35.978	1	<.001	0.047		

Table 6: Regression analysis with Justification and Country as predictors of assertability. χ^2(5,n=575)=568.09, *p*<.001, Cox & Snell R^2=.628, Nagelkerke R^2=.838. Reference classes: Justification = unjustified, Country = Japan. Coding Country (1): US=1, D=0, JP=0; Country (2), US=0, D=1, JP=0. 94.6% of cases were correctly classified.

References

Kneer, M. (2018). The norm of assertion: Empirical data. *Cognition*, *177*, 165-171.

Nichols, S., Stich, S., & Weinberg, J. (2003). Metaskepticism: Meditations in ethno-epistemology. *The skeptics*, 227-247.

Turri, J. (2018). Revisiting norms of assertion. *Cognition*, *177*, 8-11.

[5] Given that there are four possibilities – 2 assertability (yes v. no) × 2 justification (yes v. no) – the level of picking any of these by chance was ¼.
